# Supplementary material for: kmtricks: efficient and flexible construction of Bloom filters for large sequencing data collections
Source: Bioinform Adv. 2022 Apr 29;2(1):vbac029. doi: 10.1093/bioadv/vbac029 (PMC9710589; doi:10.1093/bioadv/vbac029)
Supplement: vbac029_Supplementary_Data [file vbac029_supplementary_data.pdf]

SUPPLEMENTARY MATERIALS
kmtricks: Efficient and flexible construction of Bloom filters for large sequencing data collections

Téo Lemane, Paul Medvedev, Rayan Chikhi, and Pierre Peterlongo

1 Human RNA-seq benchmarks

This section present an extensive version of the Table 1 (Section 3.2) with additional comparisons against non-Bloom filter based tools. Tool versions are shown in Table 4. Details about data and scripts are available from the `kmtricks` github companion website (see reference Lemane and Peterlongo (2022)). A Conda environment is also provided to reproduce these benchmarks.

kmer counter (& bf creation)	Index	Time (min)	Memory (GB)	Disk (GB)
**A : 100 RNA-seq (44 GB fasta.gz)**				
Jellyfish (& makebf)	HowDe-SBT	147 + 21	13.2 — 2.6	55.1
KMC 3 (& makebf)	HowDe-SBT	33 + 21	2.9 — 2.6	28.4
McCortex $k = 31$	COBS	256 + 67	27 — 1.5	327
Squeakr	Mantis	64 + 24	3.6 — 27.8	25.8
kmtricks	HowDe-SBT	24 + 21	3.6 — 2.6	45
kmtricks [R]	HowDe-SBT	26 + 21	3.4 — 2.6	46
kmtricks [R] $k = 31$	HowDe-SBT	20 + 21	3.6 — 2.6	50
**B : 674 RNA-seq (961 GB fasta.gz)**				
Jellyfish (& makebf)	∅	3543	13.2	206
KMC 3 (& makebf)	∅	1958	18.7	165
KMC 3	Metagraph	561 + 973	18.7 — 21.9	96.3
kmtricks	∅	1033	24	247
kmtricks [R]	HowDe-SBT	1060 + 120	23 — 2.4	320

kmtricks [R]: kmtricks using rescue mode

Table 1: Benchmarks on two human RNA-seq datasets of 100 and 674 samples. Computations were done using 20 threads with $k = 20$. However as `COBS` supports only `McCortex`-file for $k = 31$, we also propose results for `kmtricks` + `HowDe-SBT` using $k = 31$. For Time and Memory, when two values are provided in a cell, the first corresponds to the pre-processing time (k-mer counting and possibly Bloom filter creation) and the second to the index construction. Memory and Disk correspond to the peak usage. Disk usage corresponds to the total required space to build the index, including temporary files, Bloom filters and the final index. For `McCortex-COBS`, the disk usage corresponds mainly to the ctx files from `McCortex`.

Results not including the index creation

As shown Table ±1, on the smaller dataset (100 RNA-seq, 44 GB fasta.gz), `kmtricks` outperformed `Jellyfish` used in combination with `makebf`, `McCortex` and `Squeaker` in term of computing time (by 2.6-10x) and memory usage (by 1-3.9x) and use comparable disk space. We also substituted `Jellyfish` with `KMC3` in `HowDe-SBT`, yielding comparable time/memory performance to `kmtricks` on this collection. In terms of k-mer counting alone, `KMC3` is 1.8x faster with similar memory usage, however `KMC3` does not create Bloom filters from counted k-mers, and does not support joint k-mer counting and so can not provide a similar k-mer rescue procedure. Its integration in a Bloom filter construction pipeline turns out to be significantly less scalable than `kmtricks` as shown Section 3.3, dealing with larger and more complex data.

One the larger dataset (674 RNA-seq 961 GB fasta.gz), similar conclusions hold, `kmtricks` remaining the fastest tool to provide Bloom filters from raw read files (1.8-3.3x faster).

Results including the index creation

We used `HowDe-SBT` from Bloom filters and `COBS` and `Mantis` from counted k-mers for constructing final indexes. Except for `COBS` which is significantly longer than other tools (3.2 times longer than `HowDe-SBT`) performances are equivalent.

Even if it is currently not published, we also tested `Metagraph` (`https://github.com/ratschlab/metagraph`) on the largest dataset and using `KMC3` as prepossessing step. Compared to `HowDe-SBT` using `kmtricks` as a prepossessing step, and including the rescue mode, `KMC3` +`Metragraph` uses 3.3 times less disk, and is 1.3 times slower, while using slightly less RAM (21.9 GB versus 23 GB).

k-mer matrix construction

In this manuscript, we focused on the Boom filters construction. However, kmtricks is able to build different type of matrices like abundance or presence/absence matrices. In the table 2, we present a quick comparison between Bloom and abundance matrix construction. Since these two modes share a part of their algorithms, their performances are often close in terms of computing cost. This can of course differ depending on the parameters such as very large Bloom filter size for instance. Moreover, in Bloom mode, the fixed size of hashes allows us to use a more efficient compression algorithm, in both time and space, resulting in a less-intensive IO usage.

	**100 RNA-seq (44 GB fasta.gz)**		
	**Time (min)**	**Memory (GB)**	**Disk (GB)**
`kmtricks` Bloom	26	3.4	46
`kmtricks` k-mer	29	2.8	54

Table 2: Comparison of k-mer matrix and Bloom filter matrix construction on 100 RNA-seq samples. Computations were done using 20 threads and $k = 20$.

k-mer counting

Although `kmtricks` is not a drop-in replacement for k-mer counters, we compared it with `Jellyfish` and `KMC3` on 100 RNA-seq samples. The results are presented in the table 3. As shown in the table, `kmtricks` is faster than `Jellyfish` but it should be noted that the outputs are different since `Jellyfish` produces a hash table. For the comparison with `KMC3`, the performances are close because `kmtricks` is adapted to multi-sample counting. For single-sample counting, a k-mer counter like `KMC3` is probably more adapted and efficient.

	**100 RNA-seq (44 GB fasta.gz)**		
	**Time (min)**	**Memory (GB)**	**Disk (GB)**
`kmtricks`	26	2.2	48
`Jellyfish`	51	6.2	49
`KMC3`	24	2.9	29.8

Table 3: Comparison of k-mer counting on 1 and 100 RNA-seq samples. Computations were done using 20 threads and $k = 20$.

2 Tool versions

**Tool**	**Version or git sha1**
**HowDe-SBT**	2.00.02
**Jellyfish**	2.3.0
**KMC**	3.1.1
**McCortex**	1.0.1
**COBS**	1915fc0
**Squeakr**	0.6
**Mantis**	0.2.0
**Metagraph**	0.1.0
`kmtricks`	1.1.1

Table 4: Tool versions.

3 Empirical analysis of pBFs false positive rate

Since `kmtricks` Bloom filters are partitioned (pBFs), a potential drawback is that the partition repartition is uneven and that false positive rate is partition-dependant. We checked the false positive rate of each partition and performed the following experiment: given a pBF of total size s, we compared for each of its partitions the actual false positive rate versus the false positive rate that would be obtained by a non-partitioned Bloom filter of size s (called the theoretical false positive rate). We computed the partition-dependent false positive

rate (using 300 partitions) for a dataset with 100 human RNA-seq samples. Results shown in Fig. 1 give the false positive rate dispersion across partitions for 15 samples compared to the theoretical false positive rate of these 15 samples. Results on the remaining 85 samples are similar. Command lines and full results are available at `github.com/pierrepeterlongo/kmtricks_benchmarks`. Despite some outliers, partition-dependent false positive rates remain close to the theoretical values.

As the partitioning scheme is the same for all samples of a dataset, it is theoretically possible for some experiments (very heterogeneous for instance) and some samples that the false positive rate variation across the partitions is more important than what we observe here. For allowing query-time correction of this effect, `kmtricks` provides as an output the false positive rate of each partition for each sample.

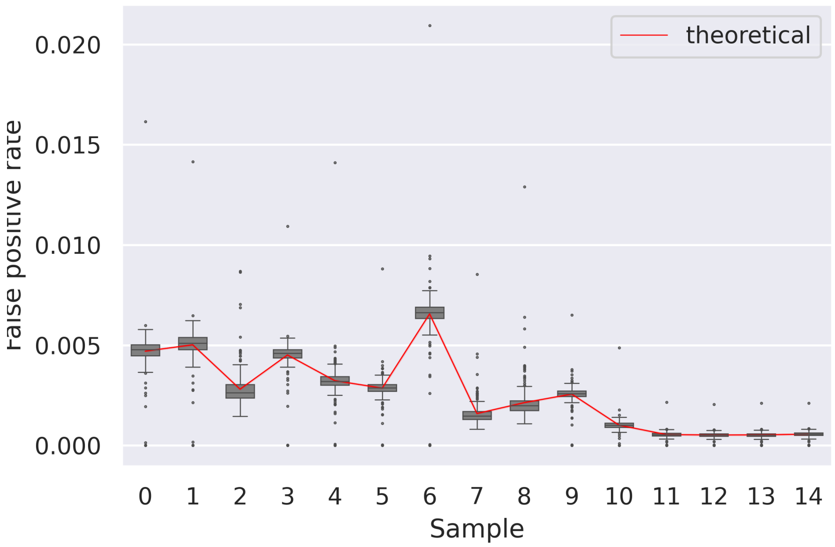

Figure 1: Partition-dependant pBF false positive rate. Given 15 human RNA-seq samples, the distribution of false positive rates across partitions is shown as well as the theoretical false positive rate, obtained with no partitioning.

4 `kmtricks` modules

`kmtricks` tool suite is composed of a set of linearly dependent modules along with some utilities and API allowing k-mer/hash/bf matrices construction. As described in Figure 2, each module corresponds to one step of the **`kmtricks pipeline`** but some can have different inputs/outputs depending on the chosen output mode (k-mer or hash counting, with or without k-mer rescue, etc...).

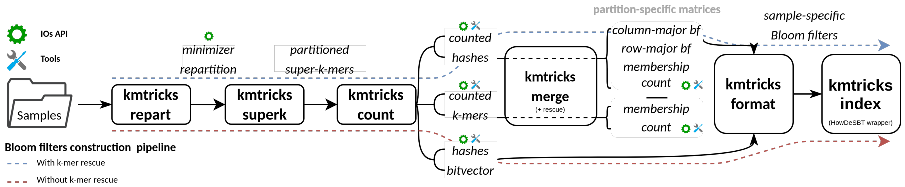

Figure 2: `kmtricks` modules overview. The different possible paths in `kmtricks`'s pipeline are represented by a diagram of modules (boxes) annotated with their intermediate outputs (italics). Many of the intermediate outputs are readable by the `kmtricks` API, and tools are also available for basic operations such as `dump` or `aggregate`. The two dotted lines show the pipeline described in the paper, i.e. Bloom filters construction with (blue) and without (red) k-mer rescue.

Additional modules are provided to exploit `kmtricks`'s files: 1) `kmtricks dump`, allowing to convert individual files in human readable format. 2) `kmtricks aggregate`, allowing to aggregated consistent files, e.g. all count sub-matrices or all counted partitions of one sample. In the same spirit, an API provides sequential reading of `kmtricks`'s files allowing for instance parallel streaming of k-mer matrices from counted k-mer partitions.
